# The Nutritional Quality of Lunch Meals Eaten at Danish Worksites

**DOI:** 10.3390/nu10101518

**Published:** 2018-10-16

**Authors:** Anne D. Lassen, Pia Knuthsen, Anette Bysted, Elisabeth W. Andersen

**Affiliations:** 1 Division of Risk Assessment and Nutrition, National Food Institute, Technical University of Denmark, DK-2800 Lyngby, Denmark; pknuan@gmail.com; 2 Research Group for Bioactives-Analysis and Application, National Food Institute, Technical University of Denmark, DK-2800 Lyngby, Denmark; anby@food.dtu.dk; 3 Danish Cancer Society Research Center, Statistics and Pharmacoepidemiology, DK-2100 Copenhagen, Denmark; elian@cancer.dk

**Keywords:** food service, nutrition, energy density, dietary intake, food and nutritional environment

## Abstract

Monitoring the nutritional environment is important to help inform future initiatives to improve access to healthy foods. The objective was to examine the nutritional quality of lunch meals eaten at 15 worksite canteens and then to compare with results from a study conducted 10 years before. The duplicate-portion-technique with subsequent chemical analysis was used to quantify 240 customers’ lunch intake. Estimated mean energy intake was 2.1 MJ/meal (95% confidence interval (CI): 1.9 to 2.4 g/meal) and estimated energy density 599 kJ/100 g (95% CI 550 to 653 kJ/100 g). Energy density of the male participants’ meals were significantly higher compared with the female participants’ meals (+55 kJ/100 g, 95% CI: +12 to +98 kJ/100 g, *p* = 0.012), whereas no gender differences were found in macronutrient distribution or fruit and vegetable intake. Compared to the study conducted 10 years before several significant changes were observed, including an increase in mean estimated intake of fruit and vegetables (+38 g/meal, 95% CI: 19 to 57 g/meal, *p* < 0.001) and a decrease in energy density (−76 kJ/100 g, 95% CI: −115, −37 kJ/100 g, *p* < 0.001). In conclusion, this study suggests an equalization of gender differences in fruit and vegetable intake and a possible improvement in the nutritional quality of canteen lunch meals over a 10-year period.

## 1. Introduction

A healthy worksite food environment should be an important goal for any organization in order to promote workers’ good health and to enhance employees’ productivity and the corporate image [1,2,3,4]. It is estimated that about one-third of our daily energy intake is consumed while at work [5], and accumulating evidence indicates that healthy food provided at work can have a significant and positive influence on our dietary intake both at work and across the whole day [6,7,8].

Successful intervention strategies at worksite canteens, restaurants, and other out-of-home outlets have included the provision of affordable healthy food options, decreasing the range of less healthy food and beverage options, some labeling schemes and different “nudging” strategies like changing the placement of fruit and vegetables [9,10,11,12,13]. These kinds of environmental-level strategies do not require the individual to self-select into a defined programme [9]. They are, therefore, more likely to target “harder to reach groups” to change dietary intake compared with relying solely on individual-level strategies [14,15,16]. Consequently, in recent years, there has been a great focus on political initiatives, as well as health programs and campaigns, aiming at supporting and stimulating health promotion initiatives in order to improve our everyday food and nutritional environment [6,17].

Despite the increasing political and scientific awareness on the importance of our food and nutritional environment, studies analyzing the availability and changes in availability of healthy food have been sparse. Lytle et al., concluded from a review on measures of the food environment that surprisingly little work have been conducted especially in the school and worksite settings [18]. An important factor for this was hypothesized to be the labor intensity of collecting and analyzing these types of data. In a recent cross-sectional study, Canterberry et al., examined 18,070 trays of food among fourth and fifth graders from seven schools and found a need to increase consumption of healthy foods, particularly fruit and vegetables among both gender [19]. In another cross-sectional analysis by Kim et al. [20], the nutritional quality of 2192 Korean workers’ lunches served by institutional or commercial food service vendors was examined. Energy intake from lunch accounted for approximately 35% of the daily energy, whereas fruit and vegetable consumption was less than one-third of the daily goal for vegetable and fruit intake in both groups. Changes over time was examined by Hearst and colleagues [21], who reported modest improvements in the average nutritional quality of menu offerings across eight fast-food restaurant chains from 1997/1998 to 2009/2010. Jarlenski et al. [22], likewise, found minor changes in menu items’ caloric and macronutrient composition from 2012 to 2014. To our knowledge, no studies have been published regarding changes in food and nutrient content of food offered at worksite canteens over time.

In Denmark, initiatives aiming at changing the food and nutritional environment during the last decade have included launching of a Keyhole labeling certification system for restaurants [13], and partnerships have been established with the goal to make healthy choices easy and accessible for all (e.g., the Meal Partnership, the Danish Salt Partnership, and the Wholegrain Partnership). Accessibility has been defined as whether available foods are in a form or location that facilitates their consumption [23]. It is not known to what extent the different initiatives have been implemented and affected the food environment and customers lunch food intake and satisfaction with the food.

The objective of the present study was to examine the nutritional quality of lunch meals consumed at 15 worksite canteens, including the intake of energy and fruit and vegetables, as well as the energy density and macronutrient distribution of the food. A further aim was to evaluate employees’ satisfaction with the meals and compare with results from a similar study conducted 10 years before.

## 2. Materials and Methods

Data collection took place between February and November in 2014. The study was performed in accordance with the ethical standards of the Helsinki Declaration of 1975, as revised in 2008. The Danish National Committee on Health Research Ethics has decided that, according to Danish Law, this kind of study does not require approval. In order to maintain participant confidentiality, anonymous identification numbers were used and participants’ names, addresses etc. was not collected in the questionnaire. Oral consent was obtained from all participants.

A total of 15 worksite canteens participating in a study conducted 10 years before [12] were contacted and asked to participate in the present study. The 15 worksite canteens had been extracted at random from a central national register representing both city and provincial towns as described in a previous paper by Lassen et al. [12]. A total of eight of the 15 worksites agreed to participate. Two worksites did not exist anymore, two worksites did not have a canteen anymore and three worksites declined to participate due to lack of time. As replacements, seven new worksites were recruited. The new worksites were recruited in the same geographical area to match the missing worksites in terms of size and occupation profile, i.e., five private financial and service organizations, eight private manufacturing and distribution organizations and two public sector organizations in both studies.

The duplicate-portion technique with subsequent chemical analysis was used to get valid estimates of nutritional quality of the lunch meals eaten at the worksites. A slightly modified method of the duplicate-portion technique [24] was used where a laboratory technician collected the duplicate plates, instead of participants collecting their own duplicate plates, in order to reduce respondent burden and to disrupt the food selection and eating pattern as little as possible. A total of 240 duplicate portions of employees’ lunch meal selections were collected over 2 days from the 15 participating canteens (a total of 16 lunch meals at each canteen).

Employees were recruited at random by asking them at specific pre-decided time-points before they selected their meals in the dining area whether they would participate in the study. A laboratory technician then followed the participants and collected identical duplicate portions of the employees’ meal selections from the buffet. A maximum of two persons at each canteen rejected to participate. Both the originals and the duplicate portions were photographed to document that portion sizes were similar between the duplicate meals and the original meals. After the employees finished eating their lunch meals, they were asked to return the plates to the technicians in order to record food not consumed, including non-edible items and edible waste. The amount of food not consumed was then removed from the collected duplicate meal portions to be able to determine the actual intake. Finally, the duplicate meal portions were individually mixed and homogenized for analysis. Beverages were not included in the analysis.

The participating employees completed a short questionnaire while photos were taken of their meals (before eating their meals) to provide background information on gender, age, weight, height, and occupation (i.e., skilled, unskilled worker, office worker, trainee, and other) (Figure S1). Also, the participants were asked about their attitudes towards healthy eating and their satisfaction with the canteen food. The question “Do you try to eat healthy foods?” could be answered as follows: “very often/always”, “often”, “sometimes”, “seldom/never”, or “don’t know”. The question “Overall, how satisfied are you with the cafeteria food?” could be answered on a 5-point rating scale from “very dissatisfied” to “very satisfied”. Body mass index (BMI) was calculated from self-reported height and weight data. Kitchen background information including the meal serving system, number of daily canteen lunch meals and overall occupation profile of the customers at the worksite according to gender distribution and proportion of customers with sedentary job functions was collected through structured interviews with the canteen managers.

Analyses of the content of protein and ash were performed according to procedures given by the Nordic Committee on Food Analysis [25,26]. Content of dry matter was determined by drying an aliquot under vacuum at 70 °C to constant weight. Carbohydrate and energy content were calculated from amounts of dry matter, protein, fat, and ash [27]. Levels of fat and fatty acids were determined according to the procedure described by Bysted et al. [28]. Recipes and methods for dish preparation were provided by the staff of the canteens, thereby providing the basis for the calculation of the fruit and vegetable content of each dish.

The same procedure was applied at the study conducted 10 years before compared to the present study, expect that whereas 240 duplicate portions of employees’ lunch meal selections were collected in the present study only 180 duplicate portions at 15 worksite canteens were collected at the study conducted 10 years before [12].

Linear mixed models were used to analyse data statistically. When comparing results according to gender the analyses were adjusted for age group, BMI group, and occupation and worksite canteen was taken into account as a random effect. The outcomes were transformed using Box-Cox transformations as this was reasonable when looking at the residual plots and then the estimates and effects were back-transformed to the original scale. When comparing results from the present study with results from the study conducted 10 years before adjustments for gender, age, BMI, and occupation were taken into account and worksite canteen was added as a random effect. The model fit was checked by residual plots and quantile-quantile (Q-Q) plots, and if necessary, Box-Cox transformation for normal distribution was performed. The estimated 10-year period effects were back-transformed to the original scale and can be viewed as covariate-adjusted differences in medians. Differences in the characteristics of the study participants between the two studies were analysed using chi2 tests for grouped variables or Fisher’s exact test if the expected number in a cell was less than 5. For continuous variables, results were compared using *t*-tests with un-equal variances.

## 3. Results

### 3.1. Characteristics of the Worksite Canteens and Study Participants

Canteen managers reported canteen customers to be mainly males by 60% of the canteens, and to have mainly sedentary job functions by 67%. A total of 33% of the canteens were outsourced to an external catering company. All worksite canteen served buffet-style, i.e., customers can directly view and select the different dishes they wish to consume, and decide how much food they take.

A total of 59% of the participants were males, 58% of the participants were 40 years or above, 39% of the participants had a BMI above 25 kg/m^2^ and 68% of the participants were categorized as office workers (Table 1). Results on participants health consciousness showed that on average, 67% of the male participants claimed that they often or very often attempted to eat healthily, compared to 88% of the female participants.

All the female participants expressed that they were satisfied or very satisfied with the canteen food compared with 92% of the male participants (Table 2).

### 3.2. Nutritional Quality of the Lunch Meals

Table 3 shows the unadjusted mean as well as the estimated mean energy content, portion size, energy density, macronutrient distribution, and content of fruit and vegetables in the meals.

Estimated mean energy density of the meals eaten was 599 kJ/100 g (95% CI 550 to 653 kJ/100 g). Estimated mean intake of total fat was 37.4 energy percentage (E%) (95% CI 33.6–41.2 E%). Estimated mean intake of saturated fat was 10.2 E% (95% CI 8.7–11.8 E%). A total of 46% of the participants consumed more than 10% of energy from saturated fat. Estimated mean intake of total fruit and vegetables was 133 g/meal (95% CI 108–161 g/meal) (unadjusted mean 161 g/meal; standard deviation (SD): 79). Vegetables accounted for 84% of the total amount of fruit and vegetables (calculated from unadjusted mean).

### 3.3. Gender Differences in the Nutritional Quality of the Lunch Meals Consumed

Table 4 shows the unadjusted mean as well as the estimated mean intake of the lunch meals consumed for female and male participants respectively, as well as the estimated median change between these groups. The average size of the meals for the male participants were significantly larger than for the female participants (+50 g, 95% Cl: +21 to +79 g, *p* < 0.001). Also, the energy density of the meals for the male participants were significantly higher compared with the meals for female participants (+55 kJ/100 g, 95% Cl: +12 to +98 kJ/100 g, *p* = 0.012). Consequently, male participants consumed more energy at lunch compared with the female participants (+492 kJ, 95% Cl: +277 to +707 kJ, *p* < 0.001). No gender differences were found with regard to macronutrient distribution or intake of fruit and vegetables.

### 3.4. Comparison of Results with a Study Conducted 10 Years Before

Compared with the study conducted 10 years before, a significant lower estimated energy intake (−205 kJ/meal, 95% CI: −383, −27, *p* = 0.024) and a significant lower estimated energy density (−76 kJ/100 g, 95% CI: −115, −37 kJ/100 g, *p* < 0.001) were observed in the present study, while no significant difference in portion size over time was seen (Table 5).

With regard to the macronutrient content, the estimated percentage of energy from protein was higher at the present study compared with the study conducted 10 years before (2.1 E%, 95% CI: 0.4 to 3.7 E%, *p* = 0.014), whereas estimated percentage of energy from carbohydrates was lower (−4.3 E%, 95% CI: −7.1 to −1.4, *p* = 0.003). Furthermore, there was a significant increase in the mean intake of fruit and vegetables compared to the previous study (38 g/meal, 95% CI: 19–57 g/meal, *p* < 0.001). Moreover, the percentage of employees with a low intake of fruit and vegetables (<100 g/meal) decreased from 47 to 19 in the present study.

No significant difference was found between the studies in percentage of energy from total fat in the meals. Likewise, the percentage of energy from saturated fat, monounsaturated fat and polyunsaturated fat were similar between the two studies.

A significant increase in numbers of male participants claiming that they often or very often attempted to eat healthily were seen in the present study compared with the study 10 years before (*p* = 0.007). Among female participants, no significant difference in attitude towards healthy eating was seen between the two studies. With regard to satisfaction with the canteen food, a significant increase was found among female participants compared with the study 10 years before (*p* < 0.001). Among male participants, no significant change in the satisfaction with the canteen food was seen.

## 4. Discussion

A high intake of fruit and vegetables is one of the cornerstones of a healthy diet [29]. In the present study evaluating the nutritional quality of lunch meals eaten at 15 worksite canteens, estimated fruit and vegetable intake was found to be 133 g per meal. An estimated increase in fruit and vegetable content by 38 g per meal was seen when comparing results with a comparable study conducted 10 years before, corresponding to a 21% increase calculated from an unadjusted mean. Importantly, the percentage of employees with a low intake of fruit and vegetables (less than 100 g per meal) decreased considerably to less than one out of five in the present study.

The intake of fruit and vegetables increased especially among male customers. This is encouraging because engaging men in health promotion is known to be a challenge [30]. Males have frequently been reported to engage less than females in health-promoting behaviours and to have less healthy lifestyle patterns [31,32]. The results from the present study suggest that men are willing to consume more fruit and vegetables if they are easily accessible and consistent with their taste preferences. Satisfaction with the canteen food was generally high in the present study and no significant change in the satisfaction with the canteen food was seen among male participants between the present study and the study conducted 10 years before. Similarly, Uglem et al. [33] concluded that the combination of increased availability of healthy food items and nutrition information was an effective way to increase the intake of vegetables among young men in the military without a reduction in food satisfaction.

Mean estimated energy density was found to be 599 kJ/100 g of the consumed meals excluding beverages. The Second Expert Report from the World Cancer Research Fund and the American Institute for Cancer Research [34] recommend the average energy density of diets to be lowered towards 520 kJ/100 g, excluding drinks, to prevent overweight, i.e., a little more than 10% lower compared to the results from this study. The lower energy density found in the present study compared with the study conducted 10 years before can probably, at least in part, be attributed to the increased intake of fruit and vegetables high in water and volume but providing less energy. Another factor known to affect energy density is the fat content. However, no significant change was observed for consumption of total fat or saturated fat between the two studies. On the other hand, a significant change was seen towards increased proportions of total energy intake from protein concurrent with a decreased proportion of total energy intake from carbohydrate. This is in accordance with trends seen in the general population in other studies, including among US adults from 1999 to 2012 [35] and among a large Swedish population-based cohort, assessing changes in intake from 1996 to 2014 [36].

Compared with the study conducted 10 years before, a significant lower estimated energy intake was observed in the present study. Worksite lunch meals provided on average 24% of daily estimated energy requirements for both men and women based on reference values given by the Nordic Nutrition Recommendations [27], assuming a sedentary lifestyle, compared to 28% and 27% 10 years before for men and women, respectively (beverages not included). This difference in energy intake can be explained by the lower energy density of the meals, while no significant difference in portion size over time was observed. Unfortunately, in the present study we do not have information on participant’s intake of beverages at lunch and also no information on whole day food intake. Therefore, we do not know to what extent the reduced energy intake at lunch was compensated for by a higher intake of calorie-dense beverages at lunch or by a subsequent higher energy intake during the day.

Although a definite explanation for changes in food consumption patterns observed in this study are unknown, a number of initiatives and programs implemented over the last decade might have contributed to an increased awareness and knowledge among restaurant professionals’ on how to cook and serve healthy meals. This includes programs such as the six-a-day fruit and vegetable campaign and the introduction of the Keyhole labeling certification system for restaurants [13]. Results showed a significant increased awareness on healthy eating among male canteen customers between the two studies, possibly leading to more healthy selections. On average, 67% of the male participants claimed that they often or very often attempted to eat healthily compared with 48% in the study conducted 10 years before. Also, the meal serving system has been shown to contribute to customers purchasing behaviors [12]. Scourboutakos et al., [37] argue that in recent years, there has been a shift toward buffet-style dining halls in universities to give students greater flexibility and more food choices. The same tendency is seen in Danish worksite canteens, and all of the worksite canteens participating in the present study had buffet-style serving, compared with about half of the canteens at the study 10 years before.

The strength of the present study includes the objective measurement of the nutritional quality of the foods eaten by the participants, using the duplicate portion technique and analysing the nutritional content by an accredited laboratory. Further, data on employees’ intake of fruit and vegetables were based on meal portions that were weighed separately rather than on self-reported information. Plate waste was taken into account and therefore the consumed food was measured instead of the served food. All canteens were visited twice as meal offerings can fluctuate in nutritional content from day to day. Finally, the survey imposed a minimum response burden on the employees, resulting in a high response rate. No significant differences in characteristics of the study participants were seen between the present study and the study conducted 10 years before with regard to gender distribution, age, BMI, and occupation type. Still, there may be other characteristics which may have influenced the nutrient intake. A limitation of the study was the relatively small sample size making it underpowered for exploration of differences between worksite types. In addition, beverages were not included in this study. Beverages, however, can make a significant contribution to the total energy intake of an individual [38].

The present results reinforce the beneficial effects of initiatives that stimulate the development of supportive food and nutritional environments. These kinds of initiatives have the power to not only influence the most health-conscious customers but also among the wider consumer. The World Health Organization (WHO) Regional Office for Europe stresses the importance of tackling social inequities in health by working with the food industry and catering enterprises to improve the nutritional quality of processed food [39]. The positive development found in the present study in relation to improved nutritional intake from canteen food should be maintained and further strengthened through continued efforts and political actions, including the further development of labelling strategies and recommendations for healthy canteen food. In line with this, a The Danish Meal Label was created in 2017 to provide guidelines to professional kitchens, e.g., at worksites, in serving nutritious food. For example, fruit and vegetables must make up at least one-third of the whole dish [40]. The guidelines contain principles that ensure the entire menu and not only the single dish. In addition, more studies on monitoring the food environment are needed. Understanding how the food and nutritional environment changes over time is important in order to study population dietary intake and to help inform future initiatives to improve access to healthy food and limit access to less healthy foods and beverages.

## 5. Conclusions

In conclusion, this study has yielded new knowledge on our food and nutritional environment at worksite canteens. Several improvements in analysed lunch intake at worksite canteens were identified compared to a previous study conducted 10 years before, including an increase in intake of fruit and vegetables and a decrease of the energy density of the canteen food. Additional findings suggest an equalization of gender differences in intake of fruit and vegetables. The results of the present study on lunch intake at worksite canteens provide a stimulus for further development, evaluation and dissemination of environmental level strategies targeting the food and nutritional environment.

## Figures and Tables

**Table 1:** Characteristics of participants.

Participants		All (*n* = 240) (%)
Sex		
	Women	41
	Men	59
Age group (years)		
	<30	16
	30–39	26
	40–49	33
	>50	25
Body mass index (BMI) (kg/m^2^)		
	<18	1
	19–25	60
	26–30	31
	>30	8
Occupation		
	Skilled	20
	Unskilled	5
	Office worker	68
	Trainee and other	6

**Table 2:** Satisfaction with the canteen food for female and male participants, respectively.

	Female (*n* = 99) (%)	Male (*n* = 141) (%) *
Very dissatisfied	0	2
Dissatisfied	0	1
Neither Satisfied Nor Dissatisfied	0	5
Satisfied	61	53
Very satisfied	39	39

* Significant difference between male and female participants in terms of satisfaction with the canteen food (*p* = 0.042, Fisher’s exact test).

**Table 3:** Unadjusted and estimated mean intake for all participants.

Nutrient and Dietary Variables	All (*n* = 240)		
	Unadjusted Mean	SD	Estimated Mean (95% CI)
Energy (kJ/meal)	2173	918	2121 (1890, 2372)
Portion size (g/meal)	371	127	352 (319, 387)
Energy density (kJ/100 g)	592	170	599 (550, 653)
Carbohydrate (E%)	40.3	12.5	40.5 (36.7, 44.3)
Protein (E%)	23.0	8.3	20.3 (18.2, 22.7)
Fat (E%)	36.6	12.1	37.4 (33.6, 41.2)
Saturated fat (E%)	9.8	4.5	10.2 (8.7, 11.8)
Monounsaturated fat (E%)	14.6	5.9	14.5 (12.7, 16.3)
Polyunsaturated fat (E%)	7.2	3.7	6.4 (5.5, 7.4)
Fruit and Vegetables (g/meal) *	161	79	133 (108, 161)

* Excluding potatoes. SD, standard deviation. CI, confidence interval. E%, energy percentage.

**Table 4:** Unadjusted and estimated mean intake according to gender and effect estimates of intake according to gender.

Nutrient and Dietary Variables	Female (*n* = 99)			Male (*n* = 141)			Male-Female	
	Un-Adjusted Mean	SD	Estimated (95% CI) Mean	Un-Adjusted Mean	SD	Estimated (95% CI) Mean	Estimated Median Difference (95% CI)	*p*-Value ^†^
Energy (kJ/meal)	1822	643	1819 (1529, 2154)	2420	1001	2313 (1962, 2714)	492 (277, 707)	<0.001
Portion size (g/meal)	333	96	308 (270, 351)	397	139	355 (312, 404)	50 (21, 79)	<0.001
Energy density (kJ/100 g)	557	167	585 (516, 660)	617	169	643 (570, 723)	55 (12, 98)	0.012
Carbohydrate (E%)	41.2	12.0	42.2 (36.9, 47.6)	39.7	12.9	41.0 (35.8, 46.3)	−1.2 (−4.7, 2.3)	0.489
Protein (E%)	22.3	7.9	20.5 (17.7, 23.8)	23.6	8.5	21.2 (18.3, 24.6)	0.8 (−1.2, 2.7)	0.458
Fat (E%)	36.5	11.6	35.5 (30.0, 41.1)	36.7	12.5	36.2 (30.7, 41.8)	0.7 (−2.4, 3.8)	0.645
Saturated fat (E%)	9.7	4.5	9.0 (7.1, 11.0)	10.0	4.5	9.5 (7.6, 11.6)	0.6 (−0.6, 1.7)	0.354
Monounsaturated fat (E%)	14.8	6.0	14.2 (11.6, 17.0)	14.5	5.9	14.0 (11.4, 16.7)	−0.3 (−1.8, 1.3)	0.747
Polyunsaturated fat (E%)	7.2	3.3	5.8 (4.7, 7.3)	7.2	3.9	5.8 (4.7, 7.2)	0.0 (−0.9, 0.9)	0.995
Fruit and Vegetables (g/meal) *	171	69	145 (114, 178)	162	86	139 (109, 172)	−6 (−25, 14)	0.559

* Excluding potatoes. ^†^ Linear mixed model adjusted for age, BMI and occupation and random effects of worksite.

**Table 5:** Estimated median change from the study 10 years before and present (estimated using linear mixed models adjusted for sex, age, BMI and education).

Nutrient and Dietary Variables	Present Study–Study Conducted 10 Years before	
	Estimated Median Change (95% CI)	*p*-Value ^†^
Energy (kJ/meal)	−205 (−383, −27)	0.024
Portion size (g/meal)	7 (−18, 32)	0.579
Energy density (kJ/100 g)	−76 (−115, −37)	<0.001
Carbohydrate (E%)	−4.3 (−7.1, −1.4)	0.003
Protein (E%)	2.1 (0.4, 3.7)	0.014
Fat (E%)	1.8 (−1.0, 4.5)	0.214
Saturated fat (E%)	0.1 (−1.0, 1.1)	0.901
Monounsaturated fat (E%)	1.2 (−0.1, 2.5)	0.078
Polyunsaturated fat (E%)	0.3 (−0.4, 1.0)	0.424
Fruit and Vegetables (g/meal) *	38 (19, 57)	<0.001

* Excluding potatoes. ^†^ Linear mixed model adjusted for gender, age, BMI and occupation and random effects of worksite.

## Supplementary Materials

The following is available online at http://www.mdpi.com/2072-6643/10/10/1518/s1, Figure S1: Demographic data collection (in Danish).
