# Supplementary material for: The Nutritional Quality of Lunch Meals Eaten at Danish Worksites
Source: Nutrients. 2018 Oct 16;10(10):1518. doi: 10.3390/nu10101518 (PMC6213244; doi:10.3390/nu10101518)
Supplement: Supplementary file 1 [file nutrients-10-01518-s001.pdf]

# Supplementary Materials

Kantineundersøgelse 2014

Danmarks
Tekniske Universitet

## Spørgsmål om dig selv og din brug af kantinen

**1. Er du mand eller kvinde?** ☐ Kvinde ☐ Mand

**2. Hvad er din alder?** ___________ år

**3. Hvad er din hovedbeskæftigelse på virksomheden?** (sæt ét kryds for hvad der passer bedst)

- ☐ Arbejder, faglært
- ☐ Arbejder, ufaglært/specialarbejder
- ☐ Funktionær/tjenestemand
- ☐ Lærling, elev
- ☐ Andet, noter venligst: ________________________________________

**4. Hvad er din højest fuldførte uddannelse?** (sæt ét kryds for hvad der passer bedst)

- ☐ Grundskole
- ☐ Studenter/HF-eksamen (inkl. HHX og HTX)
- ☐ Erhvervsfaglig uddannelse (fx håndværk, handel, kontor)
- ☐ Kort videregående uddannelse (fx social- og sundhedsassistent, merkonom)
- ☐ Mellemlang/lang videregående uddannelse (fx skolelærer, bachelor, cand.mag., læge)
- ☐ Anden uddannelse, noter venligst: ________________________________________

**5. Hvor tit spiser du typisk mad fra kantinen?** (sæt ét kryds ved hver linje)

| | 5 gange om ugen eller oftere | 3-4 gange om ugen | 1-2 gange om ugen | 2-3 gange pr. måned | Ca. 1 gang pr. måned | Ca. hver anden måned eller sjældnere | Aldrig |
|---|---|---|---|---|---|---|---|
| **Morgenmad** | ☐ | ☐ | ☐ | ☐ | ☐ | ☐ | ☐ |
| **Frokost** | ☐ | ☐ | ☐ | ☐ | ☐ | ☐ | ☐ |

**6. Hvad lagde du vægt på, da du valgte dit måltid i dag?** (sæt max 3 krydser)

- ☐ Den mad der så mest appetitlig og fristende ud
- ☐ Den mad der var mest rimelig i pris
- ☐ Den mad jeg opfattede som mest sund
- ☐ Den mad jeg plejer at spise
- ☐ Den mad som virkede mest mættende
- ☐ Madens miljøbelastning
- ☐ Noget nyt jeg ikke havde smagt før
- ☐ Jeg blev fristet af tilbud/reklameskilte
- ☐ Andet: ____________________________
____________________________

Kantineundersøgelse 2014

**7. Hvordan vil du generelt vurdere kantinens mad?** (Sæt ét kryds for <u>hver</u> linje)

| | Nej, slet ikke | Nej, i mindre grad | Ja, til en vis grad | Ja, i høj grad | Ved ikke |
|---|---|---|---|---|---|
| Maden ser appetitlig ud | ☐ | ☐ | ☐ | ☐ | ☐ |
| Smagen er god | ☐ | ☐ | ☐ | ☐ | ☐ |
| Maden er sund | ☐ | ☐ | ☐ | ☐ | ☐ |
| Prisen er passende | ☐ | ☐ | ☐ | ☐ | ☐ |

**8. I hvor høj grad er du enig i følgende udsagn?** (Sæt ét kryds for <u>hver</u> linje)

| | Meget uenig | Uenig | Hverken enig eller uenig | Enig | Meget enig |
|---|---|---|---|---|---|
| Jeg er generelt tilfreds med kantinemaden | ☐ | ☐ | ☐ | ☐ | ☐ |
| Det er nemt at finde og vælge sund mad fra kantinen | ☐ | ☐ | ☐ | ☐ | ☐ |
| Jeg ønsker mere fokus på smagen i kantinemaden | | | | | |
| Jeg ønsker mere miljøvenlig mad i kantinen | ☐ | ☐ | ☐ | ☐ | ☐ |
| Jeg ønsker mere økologisk mad i kantinen | ☐ | ☐ | ☐ | ☐ | ☐ |
| Jeg ønsker et større udbud af sundere mad i kantinen | ☐ | ☐ | ☐ | ☐ | ☐ |

Har du andre ønsker/kommentarer til kantinens mad?

**9. Bestræber du dig dagligt på at spise sundt?** (Sæt ét kryds for hvad der passer bedst)

☐ Ja, meget ofte ☐ Ja, ofte ☐ En gang imellem ☐ Nej, aldrig ☐ Ved ikke

**10. Hvor høj er du?** ______cm.

**11. Hvad vejer du?** ______kg. (For gravide: vægt før graviditet)

***Mange tak fordi du besvarede spørgeskemaet :-)***
***Besvarelsen er anonym***

**Figure S1.** Demographic data collection (in Danish).
